# Uterine dimensions in gravida 0 phase according to age, body mass index, and height in Chinese infertile women

**DOI:** 10.1097/MD.0000000000012068

**Published:** 2018-08-24

**Authors:** Hong Gao, Dong-e Liu, Yumei Li, Jing Tang, Shimin Hu, Xinrui Wu, Zhengwen Tian, Hongzhuan Tan

**Affiliations:** a Department of Epidemiology and Health Statistics, XiangYa School of Public Health, Central South University, Changsha; b School of Nursing, University of South China, Hengyang; c Reproductive Medicine Centre, Xiangya Hospital, Central South University, Changsha, Hunan, China.

**Keywords:** primary infertility, ultrasound, uterine shape, uterine size

## Abstract

The aim of this study was to describe the size and the shape of gravida-0 uteri in infertile Chinese Han women according to age, height, and body mass index (BMI).

Registered data obtained from the Department of Reproductive Medicine, Xiangya Hospital of Central South University, were collected and analyzed. The length, width, and anteroposterior diameter of the uteri of nonpregnant women aged 20 to 45 years were measured by transvaginal ultrasonography. Statistical analyses among different populations were conducted using a 1-way analysis of variance analysis or a Kruskal–Wallis *H* test.

A total of 5726 primary infertile women were enrolled. The mean age of the sample group was 29.18 ± 4.22 years, and the mean BMI and the mean height of them were 21.51 ± 2.91 kg/m^2^ and 158.13 ± 4.71 cm, respectively. The mean uterine length, width, anteroposterior diameter, and L/W ratio were 49.33 ± 7.00 mm, 39.94 ± 7.23 mm, 44.95 ± 8.11 mm, and 1.2662 ± 0.2465, respectively. There were a statistically significant positive correlations between uterine length, width, anteroposterior diameter, and age in infertile women (all *P* < .001). Uterine L/W ratio gradually decreased with age, which was statistically significant (*P* < .001). The correlations between uterine length, width, anteroposterior diameter, and height were also considered statistically significant (all *P* < .001), while there was no correlation between L/W ratio and height. The results showed that uterine size and BMI had no statistical significance.

The uterine length, width, and anteroposterior diameter gradually increased with increased age and height, but the increasing extents was different, and the uterine shape became rounder with age and had not changed with height in primary infertile women.

## 1 Introduction

The uterus is a miraculous organ that allows women to be pregnant with new life. However, a small number of women's wombs lose this function. There are many factors affecting the function of the uterus, of which the changes in size and shape of the uterus are the most important. For example, it is well-established that congenital uterine malformation can cause infertility in women. Measuring the size and shape of the uterus can help us better understand its relationship with female infertility; however, few studies concerning uterine dimensions have been published. In addition, this study was meant to explore the inherent relationships between the size and shape of the uterus and infertility.

So far, some papers have been published about the relationships between uterine size and diseases or treatments,^[1–4]^ and others described the normal uterine size in different races and ages.^[5–7]^ For example, a correlation between operation methods of hysteromyoma and uterine size had been made,^[3]^ and the normal uterine size of women in Iran had been described.^[5]^ Furthermore, an animal study found a negative correlation between uterine size and fertility in cows.^[8]^ These findings provide clarity about the importance of uterine dimensions in abnormal states of females and the differences of uterine dimensions in different races. To our knowledge, studies of uterine dimensions data of infertile women are rare in the field of reproductive health.

We aimed to describe the uterine dimensions and the relationships between age, body mass index (BMI), height, and uterine dimensions in Chinese Han women with primary infertility. We hypothesized that uterine dimensions had special changes in infertile Chinese Han women.

## 2 Materials and methods

### 2.1 Data sources

Registered data from June 2005 to May 2017 obtained from the Reproductive Medicine Centre, Xiangya Hospital of Central South University, Hunan, China, were collected and analyzed, including uterine dimensions values of primary infertile women measured by transvaginal ultrasonography. A total of 5726 infertile Chinese Han women were enrolled.

### 2.2 Study population

Women who were primary infertile women (a married woman has no contraception, with normal sex life, living together for more than 1 year, but has not yet conceive) had complete data, including uterine dimensions, age, height, weight, and BMI. In order to ensure the integrity and effectiveness of data as far as possible, we had excluded women who had abortions, premature births, induced labor, eccyesis, gravida ≥1, uterine malformations, uterine septum, uterine cancer, hysteromyoma, adenomyosis, or the uterus after surgical operation, or her husband azoospermia. The database was scrupulously checked for incomplete and incorrect values by 2 researchers.

### 2.3 Uterine dimensions measurements

Uterine dimensions of all patients were measured by transvaginal ultrasonic image examination before ART treatment. Ultrasonic scans were performed with 3.5 MHz scanners (SSD-500B; Hitachi Aloka Medical Systems, Tokyo, Japan) from 2005 to 2007 and 5.0 to 8.0 MHz scanners (DC-6 Expert; Shenzhen Mindray Bio-medical Electronics Co. Ltd., China) from 2008 to 2017 by gynecologists specialized in gynecological ultrasonography. Ultrasonic machines of ALOKA and Mindray have the same precision in measure the uterine dimensions. In our whole study, there were 3 most senior gynecologists specialized in gynecological ultrasonography, who took the uterine measurement. By 2005, they had at least 10 years of work experience. So, the uterine measurement results were reliable.

Uterine dimensions consisted of 4 main parameters: length, width, anteroposterior diameter, and L/W ratio. The length-diameter was measured from the external cervix to the fundus in the sagittal plane (another computing method: length-diameter was the sum of the cervix value and uterine corpus value, respectively, at more than c. 30°); the width-diameter was measured by the maximum diameter from the right to the left side of the uterine corpus in the transverse plane; the anteroposterior diameter was measured from the anterior serosa to the posterior serosa at the point at which the uterus appeared at its thickest and perpendicular to the endometrial line in the sagittal plane.^[7]^ Uterine L/W ratio was the uterine length-diameter divided by the width-diameter.

### 2.4 Statistical analysis

All data were managed and analyzed using the statistical package for social sciences (SPSS; SPSS Inc. 2008, Chicago, IL) software version 17.0 and Excel (Microsoft Corp., Redmond, WA). Age grouping referred to the distribution of age in the study population and similar studies^[5,7]^; height grouping referred to the statistical quartile range; BMI grouping referred to the Guidelines for Prevention and Control of Overweight and Obesity in Chinese Adults. Quantitative data were described by the mean ± standard deviation (SD) and the 95% confidence interval. The mean values of multiple samples were tested by 1-way analysis of variance (ANOVA) or Kruskal–Wallis *H* test. The variation tendency of continuous variables was described using line graph. All *P* values corresponded to 2-sided tests, and *P* < .05 was considered statistically significant.

### 2.5 Ethical approval

Medical ethics approval was obtained from the Ethics Committee of Xiangya Hospital of Central South University.

## 3 Results

### 3.1 General information

A total of 5726 primary infertile women were selected, of which 36 women's height data and 41 women's BMI values were missing. In the study population, the mean age, BMI, and height were 29.18 ± 4.22 years, 21.51 ± 2.91 kg/m^2^, and 158.13 ± 4.71 cm, respectively; the mean uterine length, width, anteroposterior diameter, and L/W ratio were 49.33 ± 7.00 mm, 39.94 ± 7.23 mm, 44.95 ± 8.11 mm, and 1.2662 ± 0.2465, respectively.

### 3.2 Correlations between age and uterine dimensions

**Figure 1:**
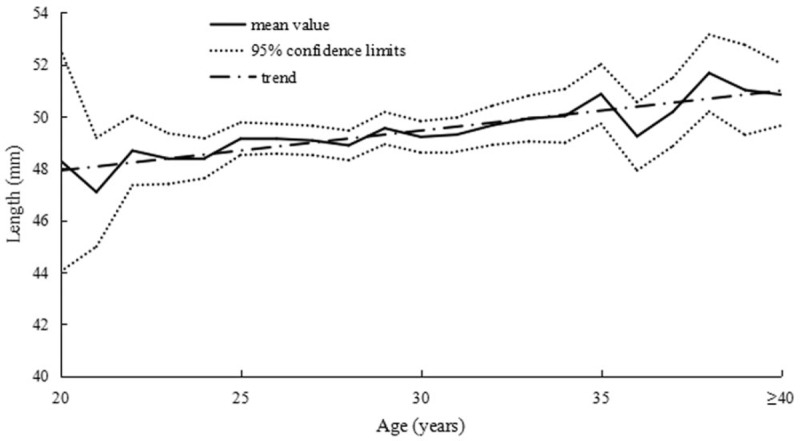
Correlation between uterine length and age in primary infertile women.

**Figure 2:**
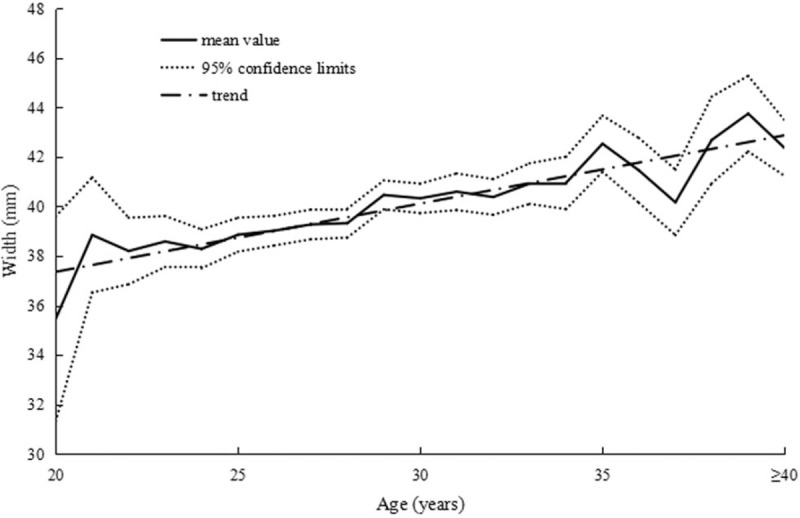
Correlation between uterine width and age in primary infertile women.

The relationships between age and uterine dimensions are summarized in Table 1. The L/W ratio gradually decreased along with the age increase from age 20 to 45 years. There were a significant positive correlations between uterine length, width, and age in infertile women (Figs. 1 and 2, respectively). As shown in the upper 2 plots, we found that uterine width increased more quickly than length. Accordingly, uterine shape became rounder with age in primary infertile women. In addition, uterine anteroposterior diameter had a positive correlation with age.

**Table 1:**
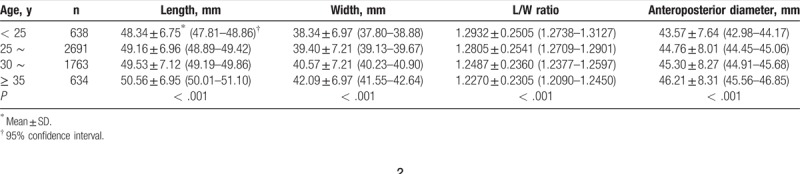
Comparisons of uterine dimensions among different age groups in primary infertile women.

### 3.3 Correlations between BMI and uterine dimensions

Table 2 summarized that all the correlations between BMI and uterine dimensions had no statistical significance.

**Table 2:**
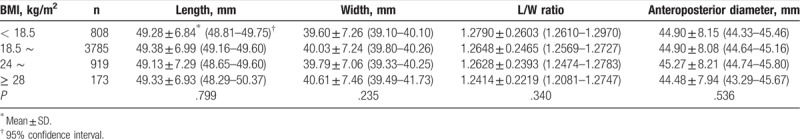
Comparisons of uterine dimensions among different body mass index (BMI) groups in primary infertile women.

### 3.4 Correlations between height and uterine dimensions

**Figure 3:**
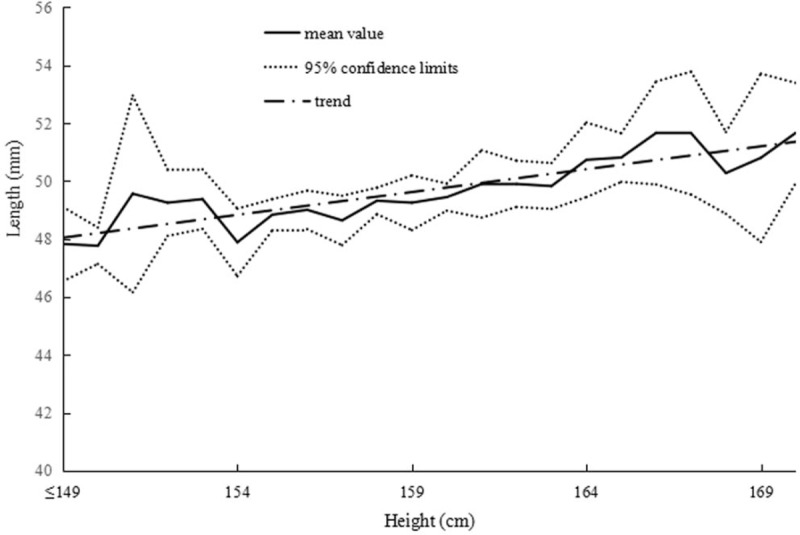
Correlation between uterine length and height in primary infertile women.

**Figure 4:**
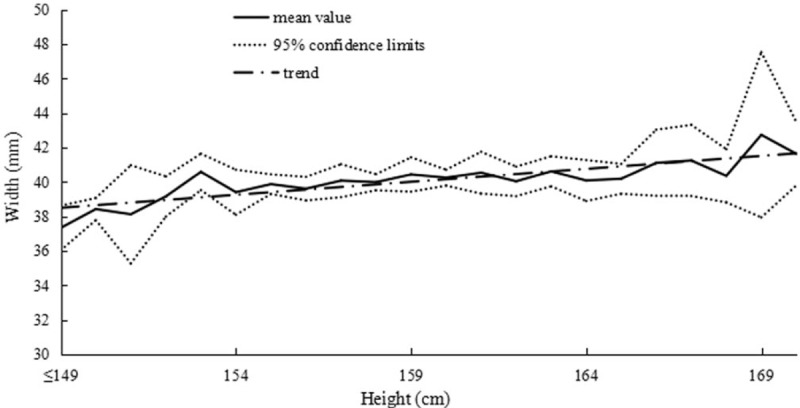
Correlation between uterine width and height in primary infertile women.

**Figure 5:**
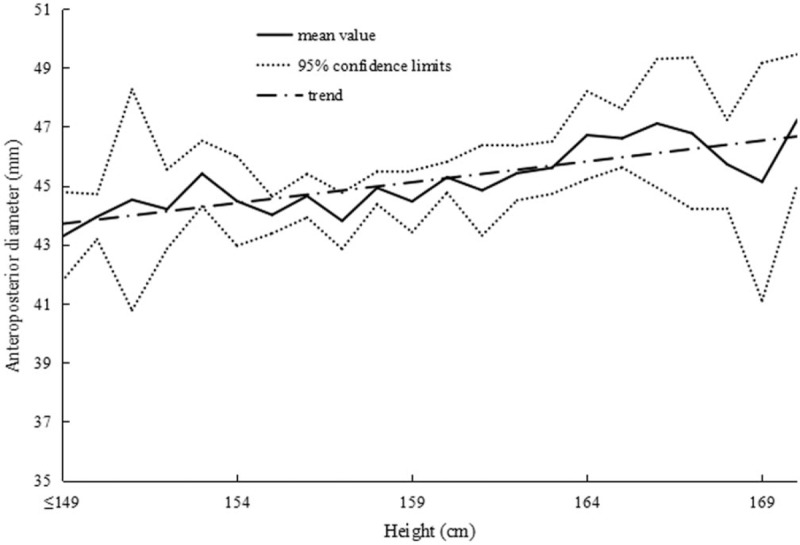
Correlation between uterine anteroposterior diameter and height in primary infertile women.

The correlations between height and uterine dimensions are summarized in Table 3. Figure 3 presents the correlation between uterine length and height in primary infertile women. In addition, 2 similar positive correlations were found between uterine width, anteroposterior diameter, and height in Figs. 4 and 5. In summary, we observed that the uterus shape had not changed with increased height in infertility in gravida 0.

**Table 3:**
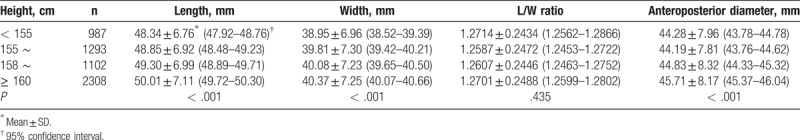
Comparisons of uterine dimensions among different height groups in primary infertile women.

## 4 Discussion

This study quantitatively described uterine size and shape in the infertile Chinese Han population for the first time. Furthermore, we analyzed the correlations between uterine dimensions and age and BMI and height. Summing up the results, we had 4 important discoveries. First, we provided objective data of uterine length, width, anteroposterior diameter, and L/W ratio in infertility with gravida-0 in the Chinese Han population. Second, uterine length, width, and anteroposterior diameter gradually increased with increased age, but the increasing extents was different: the uterus became rounder with increased age. Third, there were no relations between uterine size, shape, and BMI in primary infertile people. Finally, the uterine length, width, and anteroposterior diameter obviously increased with increased height, and the uterine shape had not changed with increased height.

In our study, the mean uterine size was 49.33 mm × 39.94 mm × 44.95 mm (length × width × anteroposterior diameter) in gravida-0 infertile women, while domestic research reported that normal nulliparous uterus size was 68.11 mm × 48.61 mm × 32.38 mm.^[9]^ This indicated that uterine length and width in infertile women in our study were shorter than those in normal nulliparous women, but the mean anteroposterior diameter was longer, making the total uterine volume of primary infertile women smaller than normal. Are there possible associations between uterine shape and volume and the infertility? Which is a valuable hypothesis and needs to be studied further.

The uterine length, width, and anteroposterior diameter gradually increased, and the L/W ratio decreased along with the age increase. However, Verguts et al^[7]^ reported completely different results, in which the mean values of uterine size in normal gravida-0 population gradually decreased with increased age. This may suggest that some other mechanisms occur in the uterus, which are related to infertility.

We found that the relationships between BMI and uterine dimensions had no statistical significance. However, other studies reported that the relationships of uterine size and BMI had positive correlations.^[2,10]^

In addition to the conclusions mentioned above, we found that uterine length, width, and anteroposterior diameter became larger with increased height in nulligravid infertile women, which were similar to other relevant researches.^[11,12]^ To our knowledge, normal uterine shape and size with height were not reported.

The strength of our study is that uterine dimensions are described objectively and quantitatively in gravida-0 infertile women. Furthermore, our sample was large enough to allow stratification of the data according to age, BMI, and height. The proportion of missing data is less than 1%.

The main limitation of the study is that we lack data for a normal control population.

The incidence of infertility increases daily,^[13–22]^ with adverse impacts on women's daily lives and marriage, which has gradually attracted more attention.^[23–29]^ The reason for infertility is very complicated. We have to step by step to understand the functions and characteristics of the various organs that affect reproduction, and to find out the causes and mechanisms of infertility. Our research is of great value and importance in the field of assisted reproduction.

## 5 Conclusion

Uterine length, width, and anteroposterior diameter significantly increased with age and height in primary infertile women, and the uterine shape became rounder with age and had not changed with height. BMI did not affect uterine shape and size. Our findings may stimulate further research on normal uterine dimensions of different races and the relationships between uterine size and pregnancy outcomes, including infertility, to determine the optimal size range of the uterus that is suitable for pregnancy.

## Acknowledgments

We would like to acknowledge the help and support of Xunjie Cheng (Department of Epidemiology and Health Statistics, XiangYa School of Public Health) and of Yanji Qu (Department of Epidemiology and Health Statistics, XiangYa School of Public Health).

## Author contributions

H.G. jointly conceptualized and designed the study, devised the linkage protocol, supervised the linkage, and carried out the analysis, drafted the initial manuscript, and approved the final manuscript as submitted. D.E.L. jointly conceptualized and designed the study, interpreted data, reviewed and revised the manuscript, and approved the final manuscript as submitted. H.Z.T. jointly conceptualized and designed the study, devised the linkage protocol, jointly supervised the linkage and carried out the analysis, reviewed and revised the manuscript, and approved the final manuscript as submitted. Y.M.L. jointly conceptualized and designed the study, interpreted data, reviewed and revised the manuscript, and approved the final manuscript as submitted. J.T. jointly conceptualized and designed the study, review data linkage, interpreted data, reviewed and revised the manuscript, and approved the final manuscript as submitted. S.M.H jointly conceptualized and designed the study, interpreted data, reviewed and revised the manuscript, and approved the final manuscript as submitted. X.R.W jointly conceptualized and designed the study, interpreted data, reviewed and revised the manuscript, and approved the final manuscript as submitted. Z.W.T jointly conceptualized and designed the study, interpreted data, reviewed and revised the manuscript, and approved the final manuscript as submitted.

**Conceptualization:** Hong Gao, Donge Liu, Xinrui Wu, Hongzhuan Tan.

**Data curation:** Hong Gao, Shimin Hu, Xinrui Wu.

**Formal analysis:** Hong Gao, Hongzhuan Tan.

**Investigation:** Hong Gao, Yumei Li, Jing Tang, Shimin Hu, Xinrui Wu, Zhengwen Tian, Hongzhuan Tan.

**Methodology:** Hong Gao, Shimin Hu, Xinrui Wu, Hongzhuan Tan.

**Project administration:** Hong Gao, Donge Liu, Yumei Li, Jing Tang, Hongzhuan Tan.

**Resources:** Hong Gao, Yumei Li, Hongzhuan Tan.

**Software:** Hong Gao, Hongzhuan Tan.

**Supervision:** Hong Gao, Donge Liu, Yumei Li, Jing Tang, Zhengwen Tian, Hongzhuan Tan.

**Validation:** Hong Gao, Yumei Li, Jing Tang, Hongzhuan Tan.

**Visualization:** Hong Gao, Yumei Li, Jing Tang, Hongzhuan Tan.

**Writing – original draft:** Hong Gao.

**Writing – review & editing:** Hong Gao, Donge Liu, Zhengwen Tian, Hongzhuan Tan.
